# Supplementary material for: Causal Relationship between Aging and Anorexia Nervosa: A White-Matter-Microstructure-Mediated Mendelian Randomization Analysis
Source: Biomedicines. 2024 Aug 16;12(8):1874. doi: 10.3390/biomedicines12081874 (PMC11351342; doi:10.3390/biomedicines12081874)
Supplement: Supplementary file 1 [file biomedicines-12-01874-s001.zip › Supplementary Figure/Figure S7/Single SNP plot of MR analysis for ubm-b-1643 to AN.pdf]

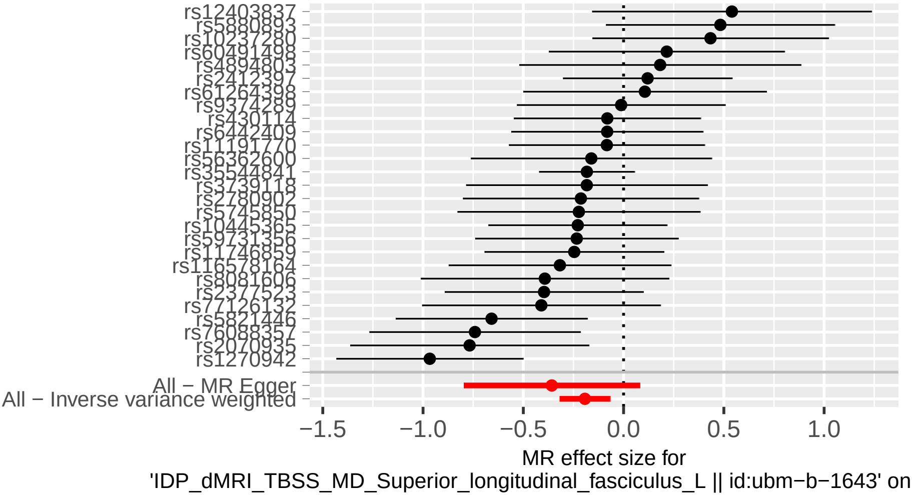

rs12403837
rs5880893
rs10237280
rs60491498
rs4894803
rs2412397
rs61264398
rs9374289
rs430114
rs6442409
rs11191770
rs56362600
rs35544841
rs3739118
rs2780902
rs5745850
rs10445365
rs59731356
rs11746859
rs116578164
rs8081606
rs2377523
rs77126132
rs5821446
rs76088357
rs2070935
rs1270942
All − MR Egger
All − Inverse variance weighted
−1.5
−1.0
−0.5
0.0
0.5
1.0
MR effect size for
'IDP_dMRI_TBSS_MD_Superior_longitudinal_fasciculus_L || id:ubm−b−1643' on
